# Abnormal expression of Krüppel-like transcription factors and their potential values in lung cancer

**DOI:** 10.1016/j.heliyon.2024.e28292

**Published:** 2024-03-21

**Authors:** Yang Shi, Min Yao, Shuijie Shen, Li Wang, Dengfu Yao

**Affiliations:** a Research Center of Clinical Medicine, Affiliated Hospital of Nantong University & Department of Medical Immunology, Medical School of Nantong University, Nantong 226001, China; b Department of Thoracic Surgery, First People's Hospital of Yancheng, Yancheng 224001, China; c Research Center for Intelligent Information Technology, Nantong University, Nantong 226019, Jiangsu, China

**Keywords:** Lung cancer, Krüppel-like factors, Zinc finger proteins, Regulatory mechanism, Tumor biomarkers, Targeted therapy

## Abstract

Lung cancer still is one of the most common malignancy tumors in the world. However, the mechanisms of its occurrence and development have not been fully elucidated. Zinc finger protein family (ZNFs) is the largest transcription factor family in human genome. Recently, the more and more basic and clinical evidences have confirmed that ZNFs/Krüppel-like factors (KLFs) refer to a group of conserved zinc finger-containing transcription factors that are involved in lung cancer progression, with the functions of promotion, inhibition, dual roles and unknown classifications. Based on the recent literature, some of the oncogenic KLFs are promising molecular biomarkers for diagnosis, prognosis or therapeutic targets of lung cancer. Interestingly, a novel computational approach has been proposed by using machine learning on features calculated from primary sequences, the XGBoost-based model with accuracy of 96.4 % is efficient in identifying KLF proteins. This paper reviews the recent some progresses of the oncogenic KLFs with their potential values for diagnosis, prognosis and molecular target in lung cancer.

## Highlights

• ZNFs/Krüppel-like factors (KLFs) refer to a group of conserved zinc finger-containing transcription factors that are involved in lung cancer progression, with the functions of promotion, inhibition, dual roles and unknown classifications.
• Some KLFs are promising oncogenic biomarkers for diagnosis, prognosis or therapeutic targets of lung cancer.

## 1 Introduction

Lung cancer still represents the main cause of tumor-related death worldwide, with the highest incidence and mortality [1,2], with a lack of early diagnostic markers, low patient survival, and poor prognosis [3,4]. The malignant neoplasms from trachea, bronchus and lung, they are classified into small-cell lung cancer (SCLC; oat cell, intermediate cell or compound oat cell type) and non-small-cell lung cancer (NSCLC; squamous cell cancer, adenocarcinoma, or large cell carcinoma, etc.) according to the degree of pathological differentiation, morphological or biological characteristics [5]. SCLC is characterized by rapid proliferation and early widespread metastasis, most of the patients with obvious hematogenous metastasis, caused by enlargement of hilar mass, huge mediastinal lymph node cough, dyspnea, and more sensitive to radio-therapy or chemotherapy [6]. NSCLC (papillary, clear cell, small cell or basal cell type), can spread along alveolar wall, local infiltration and early hematogenous metastasis, easy to involve pleural effusion. Others include adenosquamous or sarcomatoid carcinoma, carcinoid, salivary gland tumor and so on [7,8]. These data indicated that most of lung cancers originate from bronchial mucosa epithelium, but few originate from bronchial gland or alveolar epithelium.

Now, the morbidity and mortality of lung cancer are increasing rapidly, and its metastasis is the main cause of death [6]. Cancer metastasis or pathogenesis of lung cancer are complicated [9,10], and their exact molecular mechanisms remain poorly understood to be elucidated. Molecular tests have become an essential component of therapy [11,12]. Detection of epidermal growth factor receptor (EGFR) [13], epithelial-to-mesenchymal transition (EMT) mutation, exosomes, bone resorption stimulating factor (BRSF) and analysis of ALK, ROS1, RET and NTRK translocation have been incorporated into the diagnosis or treatment of lung cancer [1,5,14]. In USA, the low dose CT scan (LDCT)-based high-risk population screening protocols for lung cancer have been implemented [15], suggested that early discovery of lung cancer, with early treatment or immunotherapy might be the best approach for prolonging patients survival [[16], [17], [18]].

Recently, based on clinical and basic studies have shown that transcription factors become regulators of many key signals by affecting downstream gene expressions [19]. Among transcription factors, Zinc finger proteins (ZNFs) have been found to be the largest family in human genome, and as functional proteins involved in regulating cell differentiation, embryonic development and a variety of diseases [20]. Also, the regulation of target gene transcription factors can vary with environmental stimuli and cell types. Complex ZNFs with up to 13 Krüppel-like transcription factor (KLFs) abnormalities are related to multiple tumor progression [21]. For example, as KLF5 in normal or cancer tissues, the effects of cell growth and differentiation are very evident as transcription activators that regulate cell cycle and proliferation differentiation in lung cancer progress [22,23]; Up to date, some KLFs have been found to be involved in the proliferation, differentiation, invasion and metastasis of lung cancer. With the research progress, more and more data suggested that KLFs should be closely related to the occurrence and development of lung cancer [21,24].

## 2 KLF gene mapping & biological function

ZNFs as the largest family of transcription factors in human genome are encoded by 2 % of human genes, and have rich biodiversity functions involved in the regulation of cell differentiation, embryonic development, and the occurrence or progression of malignant tumors [19,24,25]. In addition to binding to DNA, KLFs have biological functions *via* interactions with nucleic acids or proteins [26]. According to the spatial configuration of cysteine (C, Cys) and histidine (H, His) in zinc finger, the ZNFs are divided into 8 subgroups: that is C_2_H_2_ like, zinc ribbon, Zn_2_/Cys_6_, gag knuckle, treble clef, TAZ_2_ domain like, short zinc binding loops and metallothionein [27]. Up to now, widely studied C_2_H_2_-like pattern has been confirmed that the carboxyl side of protein is linear and repetitive, contains Krüppel-associated box and poxvirus zinc finger besides their structure [28]. Among the 18 members of KLFs, the peptide chain of KLF1 ∼ KLF17 composed of 73 amino acid (a.a.) residues with 3C_2_H_2_ domains on the carboxyl side, and the zinc finger structure composed of 23–25 a.a. residues with highly conserved, has an activating/inhibiting transcription function for target molecules and can preferentially bind to 'GC box' or 'CACCC' sites, with zinc finger domains containing binding fragments to target DNA [29]. KLFs could activate or repress target genes *via* a variety of regulatory mechanisms [30]. Cys in zinc fingers is a stable, foldable, and repetitive structural protein characterized by selective binding to DNA, RNA or DNA-RNA [28,31], indicated that KLFs could play important roles in regulating gene expression, cell differentiation and related tumor development at transcription or translation level [32,33].

According to their transcriptional functions, KLFs could be divided into 3 subgroups: ① KLF3, KLF8 and KLF12 interact with carboxyl-terminal binding protein (CTBP) [33]; ② KLF1, KLF2 and KLF4 ∼ KLF7 interact with deacetylase [34]; and ③ KLF9 ∼ KLF11, KLF13, KLF14 and KLF16 inhibitors produced by corepressors Sin3A (co-Sin3A) [33,34]. The gene mapping, coding, distribution and main molecular functions of the reported KLFs are shown in Table 1 [[35], [36], [37], [38], [39], [40], [41], [42], [43], [44], [45], [46], [47], [48], [49], [50], [51]]. In addition to KLF1, KLF13, KLF14 and KLF16 or KLF18 (?) with machine learning prediction waiting for verification [52], many KLFs have been found to be associated with the progress of lung cancer by activating or inhibiting target gene expression. As above mentioned tissue-specific KLFs could induce pluripotent stem cells, and participate in immune and neovascularization processes in body, or bind abnormally to promoters to influence the biological behavior of lung cancer cells [53,54].

**Table 1:** The gene mapping, coding, distribution and main functions of the reported lung cancer-related 17 KLFs [[35], [36], [37], [38], [39], [40], [41], [42], [43], [44], [45], [46], [47], [48], [49], [50], [51]].

Name	Map	Coding	Distribution	Main function	Ref.
KLF1	19p13.13	U37106	RBC, bone marrow	Transcriptional activation	[35]
KLF2	19p13.11	AF123344	Lung, Ovary et al.	Inhibiting proliferation	[36]
KLF3	4p14	AF285837	Lung, Colon et al.	Transcriptional inhibition	[37]
KLF4	9q31.2	AF022184	Lung, Colon et al.	Transcriptional activation	[38]
KLF5	13q22.1	D14520	Lung, Skin et al.	Transcriptional activation	[39]
KLF6	10p15.2	U51869	Heart, Lung et al.	Transcriptional activation	[40]
KLF7	2q33.3	AB015132	Lung, Brain et al.	Transcriptional activation	[41]
KLF8	Xp11.21	U28282	Lung, Breast et al.	Transcriptional inhibition	[42]
KLF9	9q21.12	BC069431	Pancreas, Lung et al.	Sin3A Inhibiting	[43]
KLF10	8q22.3	U21847	Lung, Pancreas et al.	Sin3A Inhibiting	[44]
KLF11	2p25.1	AF028008	Lung, Pancreas et al.	Sin3A Inhibiting	[45]
KLF12	13q22.1	AJ243274	Brain, Lung et al.	Transcriptional inhibition	[46]
KLF13	15q13.3	AF132599	Heart, Thymus et al.	Sin3A Inhibiting	[47]
KLF14	7q32.2	AF490374	Breast, Colon et al.	Sin3A Inhibiting	[48]
KLF15	3q21.3	AB029254	Lung, Liver et al.	Transcriptional activation	[49]
KLF16	19p13.3	AF327440	Brain, Prostate et al.	Sin3A Inhibiting	[50]
KLF17	1p34.1	BC049844	Breast, Lung	Transcriptional inhibition	[51]

**RBC**, rad blood cells. **Ref**., references.

### 2.1 Regulating mechanisms of KLFs

**Fig. 1:**
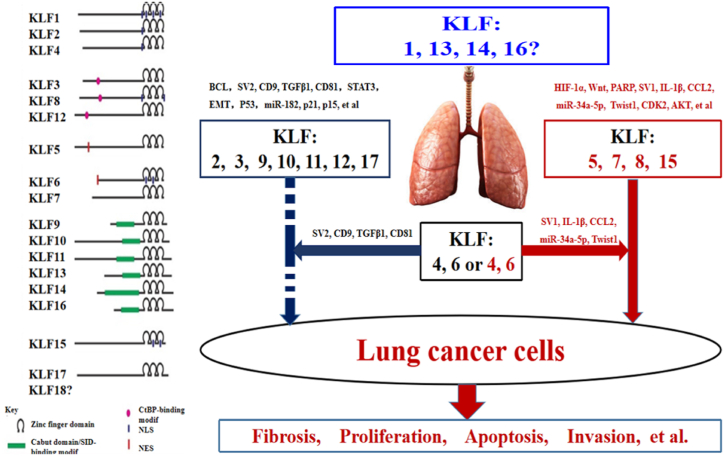
**The schematic illustration of KLFs in lung cancer**[[35], [36], [37], [38], [39], [40], [41], [42], [43], [44], [45], [46], [47], [48], [49], [50], [51], [52], [53], [54], [55], [56], [57], [58], [59], [60], [61], [62], [63], [64], [65], [66], [67], [68], [69], [70], [71], [72], [73], [74], [75], [76], [77], [78], [79], [80], [81], [82], [83], [84], [85], [86]]**.** According to the reported literature, the roles of KLFs in lung cancers were divided into four classifications with promotion (KLF5, KLF7, KLF8 and KLF15), inhibition (KLF2, KLF3, KLF9 ∼ KLF12 and KLF17), dual (KLF4 and KLF6) and unknown (KLF1, KLF13, KLF14 and KLF16) functions. KLFs refer to a group of conserved zinc finger-containing transcription factors that are involved in lung cancer develop-ment including cell proliferation, differentiation, apoptosis and so on.

KLFs and specificity protein (SP) are zinc finger-containing transcription factors, which also could play important roles in the differentiation and development of many tissues [32]. Lots of KLFs (KLF1 ∼ KLF18) and 9 SP (Sp1 ∼ Sp9) genes have been identified in human genome. Abnormality of KLFs expressions except of lung cancer are observed in various human cancers including pancreas [55], breast [56], colorectal [57], prostate [58], esophagus [59], liver [60], thyroid, stomach, bladder and cervix, which might perform different biological functions [30]. However, why are there different mechanisms and functions? Based on the reported literature, the schematic illustration of KLFs in lung cancer is shown in Fig. 1. Based on the roles in lung cancers, the KLFs were divided into four classifications with promotion (KLF5, KLF7, KLF8 and KLF15), inhibition (KLF2, KLF3, KLF9 ∼ KLF12 and KLF17), dual functions (KLF4 and KLF6) and unknown (KLF1, KLF13, KLF14 and KLF16), indicated that abnormal KLFs be derived from tissues with different biological function during lung cancer progression.

The regulatory mechanisms of C_2_H_2_-type KLFs, which have been extensively studied in the progress of lung cancer. The regulation of C_2_H_2_-type KLFs could be classified into 4 categories [27]: transcription, post-transcription, post-translational modifications (PTMs) and binding to specific DNA sequences, with its promoter CpG island methylation, suppression of related gene transcription, related miRNAs expression or environmental factors stimulate and activate cascades of related signal transduction, such as acetylation and phosphorylation, which affect the DNA binding post-translational modification of target genes [28]; protein PTMs to recruit interacting proteins [29], including transcriptional co-activators or repressors, chromosome modifiers, and other factors, binding to specific DNA sequences and activating/inhibiting downstream genes to regulate biological behaviors such as cell proliferation, apoptosis, invasion and metastasis are the main reasons for similarities and differences in lung cancer-related KLFs [30]. The data suggested that analysis of the related-KLFs be helpful to explore the new pathogenesis, immune regulation or treatment of lung cancer.

#### 2.1.1 KLFs promoting lung cancer

In lung cancer, abnormal KLFs expression could play important regulatory roles, including transcription coactivator/corepressor, chromosome modifier and other transcription factors [61], which bind to specific DNA sequences and activate downstream target genes, can regulate the proliferation, inhibit apoptosis, or promote invasion and metastasis of cancer cells [62]. Some promoting lung cancer development-related KLFs and their possible mechanism are shown in Table 2. Among the KLFs, the clear promoting effects on lung cancer were KLF5, KLF7, KLF8, and KLF15, as well as KLF4 and KLF6, which have dual functions.

**Table 2:** Promotion of lung cancer development-related KLFs and its possible mechanism^[^ [[63], [64], [65], [66], [67], [68], [69], [70]]^]^.

Name	Related genes	Possible mechanisms	References
KLF4	miR-34a-5p、IL-1β	Macrophage polarization, miR-34A-5P and IL-1β are involved in immune regulation and transcription activation	[38,63]
KLF5	HIF-1α、Cyclin B1、Survivin、Caspase 3 et al.	Promoting the proliferation, apoptosis, angiogenesis, stem cell transformation and EMT of cancer cells	[39,64]
KLF6	Twist1、CCL2、SV1	Regulation of Twist1 and CCL2 expression, macrophage polarization, and EMT promotes lung cancer cell migration	[65,66]
KLF7	miR-103/KLF7/Wnt/β-catenin, AKT/HIF-1α	Promoting the proliferation, migration and invasion of cancer cells	[41,67]
KLF8	histone demethylase, p21,CDK4	Binds to jmjd2a promoter, regulates P21 and CDK4 expression and participates in cell cycle regulation	[68,69]
KLF15	Actiating caspase-3, 7, 8,PARP	Promoting the proliferation, metastasis and anti-apoptosis of lung adenocarcinoma cells	[49,70]

**CCL2**, C–C motif chemokine ligand 2; **CDK4**, cyclin-dependent kinases 4; **EMT**, Epithelial– mesenchymal transition; **HIF-1α**, Hypoxia-inducible factor-1 alpha; **IL**-**1β**, Interleukin-1 beta; **PARP**, Poly (ADP-ribose) polymerase.

KLF4 is closely related to macrophage infiltration and polarization, and promotes the SCLC progress [38]. KLF4 expression in the cancer group is significantly higher than that in the control group, and the positive rate of stage II, III and IV is higher than that of stage I. Its expression is independent of age and sex of patients, and is a potential biomarker in late stage of SCLC [63]. KLF5 and hypoxia-inducible factors 1α (HIF-1α) in the micro-environment of lung cancer are correlated, because hypoxia can increase the activity, clonality and proliferation of A549 cells, and inhibit cell apoptosis [39]. Targeted KLF5 or HIF-1 α with specific siRNA down-regulated cyclin B1 and survivin, and up-regulated caspase-3 to promoting apoptosis are tumor promoters [64]. KLF6-SV1 is one of the KLF6 sub-types, and its expression in the adenocarcinoma group was significantly higher than that in the squamous carcinoma group, correlated with degree of differentiation, lymph node metastasis, and clinical staging [40,65], it was an independent factor affecting 5-year survival in NSCLC patients, promoting Twist1 and CCL2 expression, inducing macrophage polarization and EMT formation for lung cancer metastasis [66].

In lung cancer tissues, KLF7 was significantly higher than that in normal tissue, and associated with tumor size, lymph node metastasis, and clinical stage, and the overall survival rate of patients, indicated that KLF7 as a new prognostic marker or potential therapeutic target [41,67]. KLF8 was positively correlated with downstream target gene MMP-9, which promoted the growth, invasion and metastasis of tumor [68], with inducing VEGF expression by the PI3K/AKT pathway, and activating the Wnt/β-catenin pathway. In addition, the high expression of KLF8 enhanced the recruitment of β-catenin to T-cell factor 4 (TCF4) [42,69]. Now, the 5-year survival rate of patients with lung adenocarcinoma (LUAD) still is lower. Because tissues KLF15 was correlated with the tumor staging or differentiating degree, with higher level in cancer tissues than those in their adjacent normal tissues. However, the knockdown of KLF15 could significantly increased the expression of caspase-3, 7, 8 and the activity of DNA repair enzyme (PARP), induced apoptosis, and slowed the growth of lung xenografts in nude mice [49,70,71].

#### 2.1.2 KLFs inhibiting lung cancer

Among KLFs, several KLFs with suppressing function closely associated with the progress of lung cancer are shown in Table 3. The inhibiting roles of some KLFs (KLF2 ∼ KLF4, KLF6, KLF9 ∼ KLF12, KLF17) were detected in cancer tissues by exerting transcriptional inhibition, activating P_53_ or downstream tumor suppressor genes, inducing cell apoptosis, inhibiting key molecules of signal pathway to prevent EMT transformation, invasion or metastasis of cancer cell, etc [72]. For example, KLF2, a tumor suppressor pseudogene, was reduced in 57.5 % of lung cancer and associated with KLF2 hypomethylation and lymph node metastasis [73]. Inhibition of KLF2 by specific shRNA induced P_15_ and P_21_ expression, resulting in G_0_/G_1_ arrest of cell cycle and promoting apoptosis. High KLF2 was involved in inhibition of energy metabolism, glutaminase activity, and reduction of intracellular l-glutamine level affected lung cancer growth [74]. Bioinformatics analysis showed that KLF3 level was associated with EMT in lung cancer, with lower expression in the cancer tissues less than those in the adjacent ones. High KLF3 might decrease miR-182 expression, and its expression might restore when knockout miR-182 by 5′-azacytidine methylation inhibitors [75,76]. KLF4 expression was significantly lower in the NSCLC group than that in the normal group. Increasing KLF4 could inhibit the cell growth and promote apoptosis of lung cancer, revealing the molecular mechanism of HDACi-induced cell cycle arrest and apoptosis. CD9 and CD81 are KLF4 transcriptional targets, regulating KLF4-CD9/CD81-JNK signaling pathway and TGFβ1/SMAD signaling pathway, indicated some KLFs *via* different mechanisms play important roles in inhibiting the growth of lung cancer [77,78].

**Table 3:** Inhibiting tumor effect of lung cancer-related KLFs[[73], [74], [75], [76], [77], [78], [79], [80], [81], [82], [83], [84], [85], [86]]

Name	Target genes	Possible mechanisms	References
KLF2	p15, p21, glutaaminase	Methylation, inhibition of energy metabolism, cell cycle arrest, and promote apoptosis	[73,74]
KLF3	miR-182、STAT3	Regulating mir-182, activation of STAT3 signaling pathway	[75,76]
KLF4	CD9, CD81, TGFβ1	Inhibition of Wnt and EMT; up-regulation of Notch; induction of stem cells; activation of P53,P21 and so on	[77,78]
KLF6	SV2	Anti-cell proliferation, promote apoptosis	[40,79]
KLF9	p53	Stabilizing P53, inducing apoptosis and inhibiting Sin3A	[43,80]
KLF10	TGF-β/SMAD	Inducing apoptosis *via* TGF-β and inhibiting SP1 and Sin3A	[81,82]
KLF11	Increase in reactive oxygen species	Increasing intracellular reactive oxygen species, inducing apoptosis and inhibiting cell proliferation	[83]
KLF12	Cell cycle, anoikis, apoptosis	Cell cycle switching, regulating anoikis	[84]
KLF17	TGF-β1/Smad3	Regulating EMT, up-regulating DNA	[85,86]

**EMT**, Epithelial-mesenchymal transition; **STAT3**, Signal transducer & activator of transcription 3: **TGF-β1**, Transforming growth factor-beta 1.

KLF6 as a potential cancer cell suppressor, located at 10p15 contains three splicing variants (SV1, SV2, SV3), of which KLF6-SV2 is a tumor suppressor that exerts pro-cancer cell or anti-cell proliferation and apoptosis effects [79], respectively. As a tumor suppressor gene, KLF9 binds directly to the GC cassette in the proximal region of P_53_ promoter to stabilize p53. Its pharmacological or genetic activation has therapeutic effects, inducing G_1_/S phase arrest and cell apoptosis. Some studies *in vitro* and *in vivo* have confirmed that the mechanism could be to inhibit NF-κB activation, cell proliferation and xenograft growth [80]. Also, KLF10 (TGF-β inducible early gene 1, EGR1) is a DNA-binding transcriptional regulator with a tri-C_2_H_2_ zinc finger domain that binds to a SP1 site on DNA and interacts with other regulatory transcription factors to suppress gene expression, with anti-proliferation effect and inducing cancer cell apoptosis [81,82].

After hyperthermia combined with radiotherapy, KLF11 increased, which induced apoptosis and inhibited cell proliferation by increasing reactive oxygen species (ROS) level in lung cancer A549 cell lines. Small RNA interference with KLF11 gene transcription reduced radiothermotherapy efficacy; and the effect on lung cancer was confirmed in xenograft models, and might be a key factor in improving the efficacy of radiotherapy combined with chemotherapy [45,83]. As a new metastasis suppressor gene, KLF12 after knockout might reduced the apoptosis of lung cancer cells, and could promote S-phase cell cycle transition or regulate the anoikis. Low KLF12 enhanced the tumorigenic capacity of cancer cells and was associated with poor survival [84]. In addition, as a negative regulator of EMT and metastasis in LUAD, KLF17 regulated the Smad3-dependent pathway to enhance TGFβ1/SMAD signaling, and was a key link in anti-tumor metastasis or growth [85]. Silencing KLF17 might decrease Smad3-DNA and TGF-β1/SMAD in LUAD, that was significantly correlated with staging or size of tumors, and survival of patients [86]. Therefore, some KLFs could inhibit tumor growth *via* controlling transcription, signal molecules, EMT transformation, or activating suppressor genes and promoting cell apoptosis.

#### 2.1.3 Double or unknown function KLFs in lung cancer

According to the existing published literature, KLF4 [38,63,77,78] or KLF6 with SV1, SV2, SV3 variants [40,65,66,79] play dual roles *via* different signal molecules or pathways besides the promoting and inhibiting KLFs. However, up to now, whether some KLFs (KLF1, KLF13, KLF14 and KLF16) with unknown functions [35,47,48,50] are involved in the progressions of lung cancer have not been fully understood, and remain to be explored or verified at the levels of cell, tissue and animal models.

## 3 KLFs as potential biomarkers

### 3.1 KLFs in cancer

In the human genome, KLFs are members of the largest transcription factor family [33] that contain three distinct C_2_H_2_-type zinc finger domains at the carboxyl terminus with genetic polymorphisms, which play important roles in regulating cell proliferation, apoptosis, migration and differentiation, as well as cross-talk between signaling pathways [87,88]. Based on the accumulation of clinical and basic data [27], some KLFs were confirmed with important functions in the differentiation and development of human [89,90]. The abnormality of KLFs expression has been systematically studied in cancers of breast [91], gastric [92], prostate [93], serous ovarian [94], cervical [95], colorectal [96], and so on. Up to now, some KLFs have been identified and might become useful markers for the diagnosis or differentiation of benign and malignant tumors.

### 3.2 KLFs in benign lung diseases

The abnormal transcriptions of *KLFs* were associated with cancer progression and as biomarkers could contribute to the diagnosis or differential diagnosis of benign and malignant lung diseases, such as increased KLF5 level in pulmonary hypertension, which was associated with smooth muscle proliferation and anti-apoptosis [97]. Myeloid-specific KLF4 knockout mice, which were streptococcus pneumonia 24 h after infection. Early immune response was deficient, pro-inflammatory cytokines reduced, the anti-inflammatory cytokine IL-10 in bronchoalveolar lavage fluid and plasma was increased, bacterial clearance impaired, lung tissue damage, infection aggravated, and survival reduced. KLF4 could promote macrophage-/neutrophil-mediated inflammatory responses [98]. Also, KLF2 could be used as an early biomarker of acute lung injury [99] and involved in aerobic glycolysis and energy metabolism in NSCLC cells [74]. KLF10, which is downstream of the TGF-β/SMAD pathway, directly binds to the TGF-β receptor II promoter in CD8^+^ T cells, resulting in enhanced gene expression [100]. KLF10 gene-deficient mice are more sensitive to lipopolysaccharide or ovalbumin challenge than wild-type mice. Lung histological changes revealed increased neutrophil and severe inflammation [82], confirmed that KLF10 play an important role in the pathogenesis of chronic lung disease.

### 3.3 KLFs in lung cancer

Although the lots of diagnostic biomarkers for lung cancer, molecular tests could detect actionable genomic alterations [101,102] and cell surface proteins [103]. However, their utilization remains suboptimal, representing missed treatment opportunities for the patients. Conventional screening technologies include imaging, bronchoscopic biopsy (gold standard) and markers [5,104]. Now, serological tests such as carcinoembryonic antigen (CEA), cytokeratin 19 fragment antigen 21-1 (CYFRA 21-1), neuron specific enolase (NSE) and squamous cell carcinoma antigen (SCC), have poor specificity, with high false positive or negative rate. In this paper, KLFs have been summarized *via* systematic reviews, with either a promoting (KLF4 ∼ KLF8, KLF15) or an inhibiting (KLF2 ∼ KLF6, KLK9 ∼ KLF12 and KLF17) roles, which should be useful to monitor lung cancer progression or understand its mechanisms.

To develop sensitive and specific biomarkers for non-invasive, early lung cancer screening are particularly important, some KLFs have been studied that might have application prospects. For example, the EGR1 in NSCLC was significantly lower than that in normal lung tissues, and its expression was closely related to cell cycle arrest and apoptosis of cancer cell, with no significant association with histology and clinical stage [105]. The high EGR1 level was associated with long overall and disease-free survival of patients with NSCLC. Conversely, the low EGR1 patients were prone to relapse [81]. The level of KLF4 expression in patients at stage II, III and IV was higher than that in stage I, and might be a potential biomarker in patients with SCLC [64]. However, clinical data in this area are scarce and require large samples, multicenter validation, and future accumulation.

## 4 KLFs for prognosis

LUAD is a common form of NSCLC, with a 5-year survival rate under 15 %. The abnormal KLFs gene transcription in the tissues or circulating blood of lung cancer might be helpful to evaluate the prognosis of patients. Bioinformatics analysis showed that KLF3 level was associated with prognosis by EMT, the epigenetic silencing KLF3 mRNA increased the pro-metastatic miR-182 expression, with the mechanism of lung cancer metastasis dependent on activating STAT3 signaling pathway [76]. The KLF3 expression in lung cancer was lower than that in adjacent tissues, which was associated with poor prognosis and TNM stage [77]. High KLF7 expression in LUAD was related to tumor size, lymph node metastasis, staging, low overall survival, and an independent prognostic factor [42,106]. The mechanism of the KLF7 expression revealed that the STAT3-induced linc 00668 up-regulating KLF7 *via* spongy miR-193a [107,108] to promote NSCLC progression, and high KLF7 in LUAD with unfavorable clinical outcomes.

Some KLFs affect the prognosis of patients with lung cancer *via* complex regulatory mechanisms. KLF4 with macrophage infiltration and polarization in lung cancer microenvironment was closely associated with macrophage M2 polarization promoting cancer cell growth and survival [38,109]. In LUAD, high KLF15 level correlated with staging and differentiation degree. However, knockout of KLF15 significantly increased the levels of caspase-3, 7, 8 and DNA repair enzyme (PARP), and induced apoptosis. Down-regulated KLF15 in A549 cells or NCI-HL650 cells resulted in significantly slower growth of xenograft tumors [110] and could be a potential therapeutic target or prognostic marker for LUAD. Some KLFs could inhibit the proliferation, migration, invasion and EMT of lung cancer cells, with the prognostic value or potential markers of diagnosis and treatment for lung cancer. Although specific KLFs might serve as useful molecular markers for the diagnosis, prognosis or treatment of lung cancer, the exact regulatory mechanism of KLFs remains to be explored in the future.

## 5 KLFs with cancer stem cells (CSCs)

Recent progress has highlighted the significance of KLFs in tumor progression and CSCs. The regulatory functions of KLFs in the development of lung cancer and CSCs have become a burgeoning area of intense research. CSCs are a subpopulation of cancer cells that play critical roles in tumor propagation, therapeutic resistance, metastasis, and recurrence. CSCs have been identified to be responsible for such malignant properties of cancer as phenotypic heterogeneity, chemoresistance and dormancy [111]. Highly conserved ZFP/KLFs play anti-proliferative or pro-proliferative roles in cancer by regulating various gene expressions. In lung cancer, CSCs are rare tumor-mass cells that are thought to be responsible for recurrence, drug resistance and metastasis [31]. However, little is known about the mechanisms that regulate the differentiation and self-renewal of CSCs. KLFs are closely associated with lung cancer progress and important DNA-binding transcription regulators with multiple functions in a variety of cellular processes, including differentiation, proliferation, inflammation, migration and pluripotency [112]. The regulation of xenograft tumors in tumor-bearing mice during CSC development has become a new hotspot [113]. Also, KLFs maintain the pluripotency of CSCs by regulating cell proliferation, differentiation, development and regeneration, with bioinformatics evidence that KLFs directly regulate stem cell genes [54,114], because KLFs are essential DNA-binding transcriptional regulators with diverse functions in various cellular processes.

In the embryonic stem cell subnucleus, KLF2 and KLF4 co-localize with OCT4 [28], and KLF-DNA binding dynamics occur during differentiation for direct regulation of stem cell genes and tumor neovascularization [115,116]. The double life of KLF5 promoter region interacts with enhancer and transcription start site of colon cancer-associated transcription 1 gene, with heterologous deletion impairs cancer stem identity, bromodomain containing 4 and other cofactors participates in a core regulatory pathway, constructing a three-dimension structure (promoter, enhancer and transcription start sites), which plays opposing roles in regulating gene expression, cellular function, and transformation [117]. Interestingly, a novel computational approach has been proposed by using a noninvasive machine learning on features calculated from primary sequences, the XGBoost-based model is efficient in identifying KLF proteins, with accuracy of 96.4% [118]. The genetic algorithm plus XGBoost classifier exhibited the favorable performance, with an accuracy of 0.836 for EGFR or 0.86 for KRAS mutations in NSCLC by including the least number of the most semantic radiomics features [119]. Although much has been explored about the functions of KLFs in lung cancer [120,121], suggesting that KLFs might be an important regulator of many CSC genes, and the special effect of each KLFs in mediating CSC functions still remains to be uncovered.

## 6 KLFs for target therapy

### 6.1 Epigenetics alteration

A systematic role of metabolomics, metabolic pathways, and abnormal transcription of lung cancer-associated KLFs gene in tissues are helpful for targeted therapy of lung cancer [122,123]. MicroRNA regulation promoted suppression of lung cancer cell metastasis by acting on KLFs [37,48] and transcriptional repressor KLF3 regulated miR-182 expression that was increased after knockout of the KLF3 gene using methylation-specific PCR and pyrosequencing techniques, KLF3-DNA hypermethylation and apparent silencing. After using DNA methylation inhibitor 5′ azacytidine restored KLF3 expression, while miR-182 expression was reduced. Methylated drugs could modulate miR-182 expression *via* KLF3 as a potential target strategy for lung cancer [75]. The suppressor gene KLF2 methylation was significantly reduced in NSCLC because of its region 4 methylation associated with lymph node metastasis and advanced tumor stage. Up-regulating KLF2 could inhibit NSCLC cell proliferation, induce P15 and P21 expression to arrest the G0/G1 cell cycle and promote apoptosis [73], indicated that KLFs might be regulated through epigenetic alterations to target lung cancer.

### 6.2 Signaling pathways

**Fig. 2:**
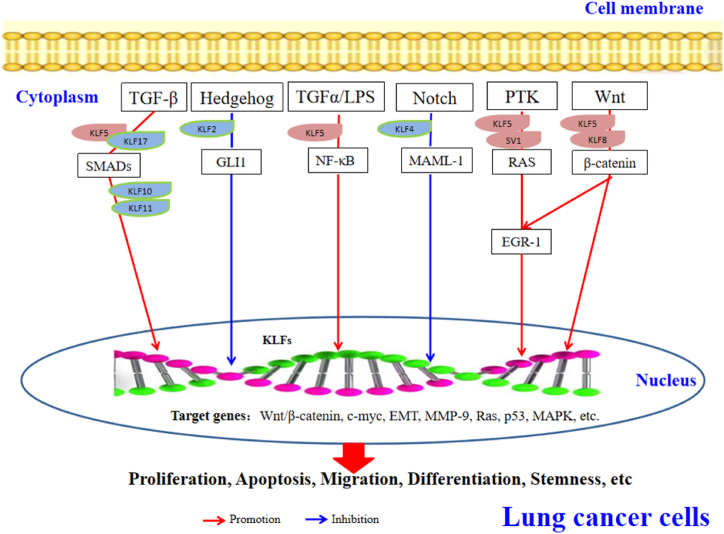
**Interaction between some KLFs and important signaling pathways.** Based on the reported literature, KLFs play roles in the promotion and inhibition of lung cancer progression through related signaling pathways. In particular, KLF4 and KLF6 could play dual roles *via* different signaling molecules or pathways (details in Table 2 or Table 3), and participate in the proliferation, differentiation and apoptosis of lung cancers.

The expressions of lung cancer-associated KLFs were related to single or multiple signal pathways *in vivo* (Fig. 2). For example, the abnormality of KLF4 expression inhibited the growth and invasiveness of NSCLC by promoted apoptosis, regulating related to the TGF-β1-meidated ERK/JNK/NF-kB pathways [78]. Placental specific 8 (PLAC8) was positively correlated with tumor size, histological grade, TNM stage, and poor prognosis of lung cancer. However, the KLF4 could negatively regulate PLAC8 promoter to exert transcriptional repression. So the KLF4/PLAC8 axis should be a candidate targeted therapy for lung cancer [124]. In addition, KLF10 contains a triple C_2_H_2_ zinc-finger domain, which is a DNA-binding transcriptional regulator that binds to specific protein 1 (SP1) site by the TGF-β/SMAD pathway [125]. And KLF17 was positively associated with Smad3, and could create a positive feedback loop [85]. Silencing *KLF1*7 might reduce Smad3- DNA complex formation as a novel model for regulating the TGF-β/SMAD pathway to inhibit the growth of A549 or PC-9 cells and to insight into anti-metastatic function [86,126], suggested that these oncogenic KLFs might be promising molecular target or combining with immunotherapy for lung cancer therapy [127,128].

## 7 Prospect

In summary, as the largest KLF families are widely involved in a variety of biological processes in the human body, regulating downstream gene transcription, participating in cell proliferation, apoptosis, invasion and metastasis, playing a pro-/anti-cancer role. Although oncogenic KLFs transcription is closely related to lung cancer progress, due to the variety and complex structure of KLFs, the current understanding was only in cell level, human tissues or animal models. A better understanding of regulatory mechanism of some KLFs might provide potential molecular markers for diagnosis, prognosis or therapeutic strategies for patients with lung cancer. However, there still is a long way to go before fully understand its regulatory functions and single KLF or few KLFs with redundant functions in CSCs. In addition, recent progress has also highlighted the importance of microenvironment for the initiation and maintenance of CSCs. With further elucidation of the KLFs implication in lung cancer, to achieve utility of KLF transcription regulators as targets will be expected for diagnostic biomarkers or therapeutic interventions.

### 7.1 Search strategy and selection criteria

Data for this review were identified by recent searches of Medline, PubMed, and references from relevant articles using search terms “Lung cancer”, “Krupple-like Factor”, “Promoting role”, “Inhibiting role”, “Regulatory mechanism”, “Tumor biomarkers”, “Diagnostic value” “Prognostic value”, and “Targeted therapy”. Only published articles in English were included.

## CRediT authorship contribution statement

**Yang Shi:** Writing – original draft, Visualization, Formal analysis, Conceptualization. **Min Yao:** Writing – original draft, Validation, Funding acquisition, Formal analysis, Data curation, Conceptualization. **Shuijie Shen:** Writing – original draft, Formal analysis, Conceptualization. **Li Wang:** Writing – review & editing, Writing – original draft, Supervision, Project administration. **Dengfu Yao:** Writing – review & editing, Writing – original draft, Supervision, Funding acquisition.

## Declaration of competing interest

The authors declare no conflict of interest.
